# Dietary Sodium Intake and Risk of Cardiovascular Disease: A Systematic Review and Dose-Response Meta-Analysis

**DOI:** 10.3390/nu12102934

**Published:** 2020-09-25

**Authors:** Yi-Jie Wang, Tzu-Lin Yeh, Ming-Chieh Shih, Yu-Kang Tu, Kuo-Liong Chien

**Affiliations:** 1 Institute of Epidemiology and Preventive Medicine, College of Public Health, National Taiwan University, No.17, Xu-Zhou Rd.,Taipei City 10055, Taiwan; r08849021@ntu.edu.tw (Y.-J.W.); 5767@mmh.org.tw (T.-L.Y.); littlecanargie@gmail.com (M.-C.S.); yukangtu@ntu.edu.tw (Y.-K.T.); 2 Department of Family Medicine, Hsinchu MacKay Memorial Hospital, No. 690, Section 2, Guangfu Road, East District, Hsinchu City 30071, Taiwan; 3 Department of Internal Medicine, National Taiwan University Hospital, No. 7, Zhongshan S. Rd., Zhongzheng Dist., Taipei City 10002, Taiwan

**Keywords:** cardiovascular disease, 24 h urinary sodium excretion, sodium intake, dose-response meta-analysis, cardiovascular mortality

## Abstract

Dietary sodium intake has received considerable attention as a potential risk factor of cardiovascular disease. However, evidence on the dose-response association between dietary sodium intake and cardiovascular disease risk is unclear. Embase and PubMed were searched from their inception to 17 August 2020 and studies that examined the association between sodium intake and cardiovascular disease in adolescents were not included in this review. We conducted a meta-analysis to estimate the effect of high sodium intake using a random effects model. The Newcastle-Ottawa Scale assessment was performed. A random-effects dose-response model was used to estimate the linear and nonlinear dose-response relationships. Subgroup analyses and meta-regression were conducted to explain the observed heterogeneity. We identified 36 reports, which included a total of 616,905 participants, and 20 of these reports were also used for a dose-response meta-analysis. Compared with individuals with low sodium intake, individuals with high sodium intake had a higher adjusted risk of cardiovascular disease (Rate ratio: 1.19, 95% confidence intervals = 1.08–1.30). Our findings suggest that there is a significant linear relationship between dietary sodium intake and cardiovascular disease risk. The risk of cardiovascular disease increased up to 6% for every 1 g increase in dietary sodium intake. A low-sodium diet should be encouraged and education regarding reduced sodium intake should be provided.

## 1. Introduction

Noncommunicable diseases (NCDs) including hypertension and cardiovascular disease (CVD) are the leading cause of mortality and morbidity globally [1]. According to the World Health Organization, NCDs account for approximately 41 million deaths each year, and account for 71% of the overall total global deaths annually. A total of 17.9 million deaths are attributable to CVD annually, and CVD accounts for 44% of the deaths from NCDs [2]. The major sources of salt are processed foods, ready-made meals and salt added during food preparation and cooking, and at the table [3]. Salt is a combination of sodium and chloride. Table salt contains approximately 40% sodium by weight. Excessive sodium intake increases blood pressure (BP), and hypertension is a risk factor for CVD [4]. In the INTERSALT study, an international study of salt and BP, BP had a positive association with 24 h urinary sodium excretion in different regions of the world [5]. The relationship between BP levels and CVD risk in adults has been established in the literature [6], and sodium reduction policies have become the main strategy to deal with the prevention of CVD. The World Health Organization recommends a daily sodium intake of less than 2 g [1,7]. The 2015–2020 U.S. Dietary Guidelines suggest that the dietary sodium intake of adults should not exceed 2300 milligrams [8]; however, a sodium intake of no more than 1500 mg per day is recommended [9]. In a meta-analysis by He et al., reducing salt intake for more than four weeks was found to have a significant beneficial impact on lowering BP. In patients with high BP, the mean change in urinary sodium excretion was 75 mmol per 24 h, with a mean reduction of 4.18 mmHg in systolic BP and 2.06 mmHg in diastolic BP [10]. These results were in agreement with two meta-analyses [11,12]. Indeed, in a dose-response meta-analysis performed by Graudal et al., a significant relationship between sodium reduction and BP was observed in the highest 25th percentile of the population [11]. Furthermore, Huang et al. and colleagues conducted a meta-analysis to evaluate the effect of dietary sodium reduction on BP [12]. The study demonstrated a dose-response relationship between BP and sodium reduction, especially in participants with high BP. For every 50 mmol decrease in the 24 h sodium excretion, systolic BP decreased by 1.10 mmHg and diastolic BP decreased by 0.33 mmHg. However, there was no correlation between the duration of sodium reduction and BP. Moreover, a systematic review showed a significant positive relationship between sodium excretion and BP [13]. Twenty-four hour urinary excretion is the gold standard method for sodium assessment. The excretion of sodium in urine accounts for about 90% of total sodium excretion [10]. Campbell et al. found that for approximately 93% of the population, sodium intake could be accurately assessed by twenty-four-hour urinary excretion. However, it is appropriate to collect multiple 24 h urinary samples for a long period of time to assess participants’ usual long-term sodium intake [14].

High sodium intake has been associated with high CVD mortality in the general population [15]. However, a prior study reported that lower sodium excretion was associated with higher CVD mortality [16]. Furthermore, a review by Taylor et al. showed no clear evidence of the benefits associated with salt reduction [17]. Indeed, the effects of sodium intake on global CVD events are unclear. To clarify the relationship between sodium consumption and CVD risk, this meta-analysis investigated cohort studies in order to summarize the epidemiological evidence regarding dietary sodium intake and risk of CVD.

## 2. Materials and Methods

### 2.1. Data Sources and Literature Searches

This dose-response meta-analysis was conducted in accordance with Preferred Reporting Items for Systematic Reviews and Meta-Analyses(PRISMA) guidelines, which are a set of preferred reporting items for systematic reviews and meta-analyses (Supplementary S1) [18]. We performed an initial search in PubMed and Embase to identify keywords contained in the title or abstract and index terms used to describe the relevant terms in the articles. We used the keywords listed as follows: (cardiovascular disease) AND (sodium intake) OR (urinary sodium excretion) AND (cohort studies) AND (human). Detailed search strategies are shown in Supplementary S2. To avoid missing the studies that met our criteria, we conducted a manual search by checking the reference lists of studies included in previous meta-analysis papers on dietary sodium intake and CVD risk [19,20,21]. Embase and PubMed were searched from their inception up to 17 August 2020.

### 2.2. Study Selection

Inclusion criteria included: (i) population over 18 years old without CVD; (ii) general population with CVD history at baseline and adjusted CVD history in statistical analyses; (iii) evaluation of the association between dietary sodium intake and risk of CVD; (iv) the endpoints were the incidence and mortality of combined or single CVD events including stroke, coronary artery disease and heart failure; (v) cohort study design; and (vi) full-texts in English. Duplicate publications, irrelevant articles, case-control studies, cross-sectional studies, randomized controlled trials, articles that provided a sodium-potassium ratio, sodium-creatinine ratio or sodium-calories ratio but not dietary sodium intake were excluded.

Two independent reviewers (YJW and TLY) identified studies based on the information in the title, abstract and description. Group discussion was conducted to resolve any disagreement between the two reviewers. Full texts were retrieved if the studies fulfilled the inclusion criteria. If eligibility of an article was unclear, two independent reviewers (YJW and TLY) would retrieve the article for further clarification based on the title, abstract and description. When relevant information regarding the design or outcomes was unclear, the original authors of the article were contacted for further clarification.

### 2.3. Data Extraction and Quality Assessment

Two independent reviewers (YJW and TLY) extracted the following study-level characteristics from each eligible study: first author, year of publication, the study region, participant characteristics, the number of person-years and incident cases, levels of sodium intake in all groups, measurement of sodium intake, adjusted variables, and rate ratio (RR) estimates with 95% confidence intervals (CIs) for each group. Two reviewers separately assessed the methodological quality of each study by using the Newcastle-Ottawa Scale [22]. We conducted a group discussion to resolve any discrepancy between the two reviewers (YJW and TLY). The checklist included eight items on three topics: (i) selection of study participants; (ii) comparability of study groups (iii) assessment of outcome and exposure. Regarding the selection and outcome, a study was awarded a maximum of one star for each question. With regard to the comparability of the study groups, a study was awarded a maximum of two stars. Each study could receive a total of nine stars for the three topics. The quality scores were based on the total number of stars.

### 2.4. Data Synthesis and Analysis

For the measurement of the relationship between dietary sodium intake and the risk of CVD, RR with 95% CIs were used. First, we conducted a meta-analysis for the highest versus the lowest category of dietary sodium intake using a random effects meta-analysis that accounted for within- and between-study variation [23]. Second, we analyzed the dose-response meta-analysis by using the reported mean of each dietary sodium intake category. The average value of the lower and upper bounds of each category was used when the studies reported the ranges of sodium intake categories. If the lowest category was open ended, we used the average value of the upper bound and zero. If the highest category was open ended, the average value was assumed to be 1.2 times the lower boundary [24].

We used the Greenland and Longnecker method to estimate the linear trends in the association between dietary sodium intake and risk of CVD [25,26]. We pooled the estimated linear trends by random-effects meta-analysis. Next, for the nonlinear trends, we first estimated a nonlinear relationship between dietary sodium intake and risk of CVD within each study by using the restricted cubic splines model with three knots (percentiles of 10%, 50% and 90%) [27]. The results obtained for each study were then pooled together by using random-effects multivariate meta-analysis [28,29], known as the two-stage approach. We performed a likelihood ratio test to evaluate the difference in model fit between the linear and nonlinear models [30]. To examine the two slopes in the non-linear model, the Wald test was used to test linearity. We used the method described by Hamling and colleagues to recalculate the log risk ratios and associated standard errors if the lowest category was not the reference category [31]. The primary outcomes were the highest versus the lowest and dose-response meta-analysis between dietary sodium intake and total CVD risk.

We assessed the heterogeneity between studies with the Q test and I2 statistics. If the values of I2 were below 25%, they were considered low, those between 25% and 75% were considered moderate, and those higher than 75%, were considered high [32].

A subgroup analysis was conducted to explain the observed heterogeneity. We calculated pooled effect estimates by follow-up years (<10 years or ≥10 years), study regions (Asia, non-Asia or multi-nation), sodium assessment (diet or urine) and types of outcomes. In the meta-regression, we assessed the following factors: mean age, women percentage, follow-up duration, percentage of white participants, body mass index and quality scores. We performed a sensitivity analysis in order to examine the robustness of our findings by omitting one study at a time and re-examining the pooled estimates. We assessed the publication bias by funnel plots. Egger’s test was used to evaluate symmetry [33]. A *p* < 0.05 was considered statistically significant. The data were analyzed using R software version 1.1.456, with “readxl”, “doresmeta”, “meta”, “metafor”, “mvtnorm”, “mvmeta” and “rms” packages.

## 3. Results

### 3.1. Study Characteristics and Quality Assessment

**Figure 1:**
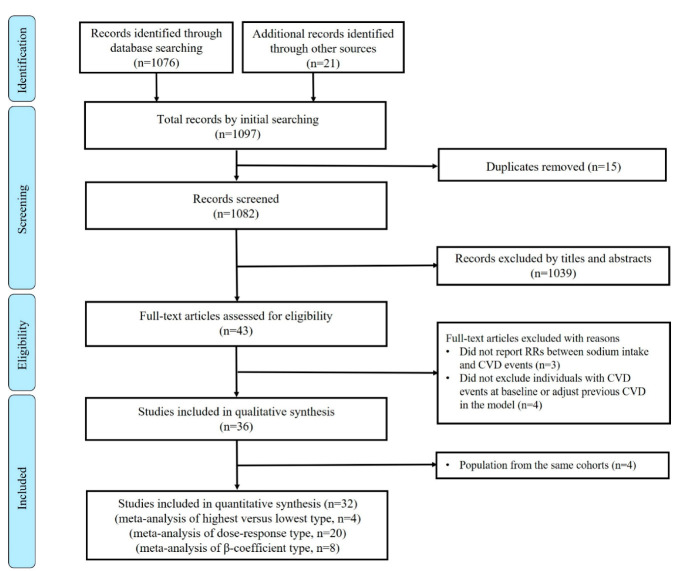
Flow chart of the literature search for studies investigating association between dietary sodium intake and the risk of cardiovascular disease. Abbreviation: CVD, cardiovascular disease; RRs:Rate ratios.

We identified 1097 articles, of which 1054 articles were excluded based on duplicate articles, titles and abstracts. A total of 43 articles were eligible for a full-text review. Among the 43 articles reviewed, 36 [34,35,36,37,38,39,40,41,42,43,44,45,46,47,48,49,50,51,52,53,54,55,56,57,58,59,60,61,62,63,64,65,66,67,68,69] met all the inclusion criteria (Figure 1). Twenty-four studies were included for highest versus lowest meta-analysis between dietary sodium intake and CVD risk. For the dose-response meta-analysis, we excluded 4 studies [35,49,62,68] because they only compared the highest and lowest sodium intake categories. After estimating the trends in the dose-response meta-analysis, eight studies reported coefficients between dietary sodium intake and CVD risk, which were added to the studies in the dose-response meta-analysis. A total of 28 studies were analyzed for subgroup analysis and meta-regression to explain the observed heterogeneity of the studies.

A list of studies included after a full-text review are presented in Table S1. Fifteen studies were based in the USA, 9 were from Europe, 9 were from Asia, and 3 were from multiple countries. The duration of follow-up ranged from 2.7 years to 29 years. Most studies included both men and women, however, one study used a female-based population [63], whereas two only included men [34,45]. For dietary sodium intake assessment, eighteen of the studies were based on twenty-four urinary excretion, seven used food frequency questionnaires, seven used 24 h dietary recall, two used dietary records; one used a self-administered questionnaire and one used single spot urine sodium excretion. The outcomes of the studies included total CVD, CVD mortality, stroke, coronary heart disease, myocardial infarction and heart failure. The daily dietary sodium intake ranged from 1.0 to 7.5 g.

The mean score (± standard deviation) for the quality of the included studies was 8.0 ± 1.0 according to the Newcastle-Ottawa Scale (Table S2). Most of the included studies had a quality score higher than 7. Hu and colleagues published the study with the lowest score of 5, as they only adjusted for age in the study, and assessed sodium intake by using a household survey questionnaire. The subjects in this study were followed up for only 4 years [35]. Two studies were scored 6 and one of those studies recruited patients undergoing peritoneal dialysis who were not representative of the general population and did not exclude patients with CVD at baseline. Additionally, the follow up duration was only 31.4 months [47]. The other study determined CVD by using self-reported intake of drugs and assessed sodium intake by spot urine collection with a loss follow-up rate higher than 20% [56].

### 3.2. Highest Versus Lowest Meta-Analysis

**Figure 2:**
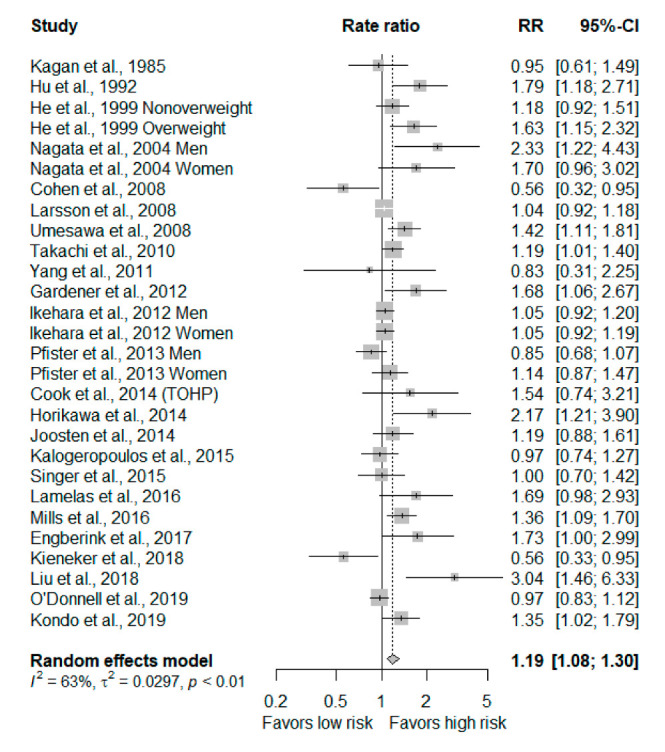
Forest plot of total cardiovascular disease^1^ for highest versus lowest categories of dietary sodium intake in 24 studies. 1: Total cardiovascular disease. This included total cardiovascular disease, cardiovascular disease mortality, stroke, coronary heart disease, myocardial infarction and heart failure. Abbreviation: RR: Rate ratios;CI: confidence interval.

In terms of CVD risk, a total of 24 studies were included in the high versus low meta-analysis. The results indicated a higher risk for individuals with high sodium intake (RR, 1.19; 95% CIs: 1.08–1.30; Figure 2).

### 3.3. Dose-Response Meta-Analysis

**Figure 3:**
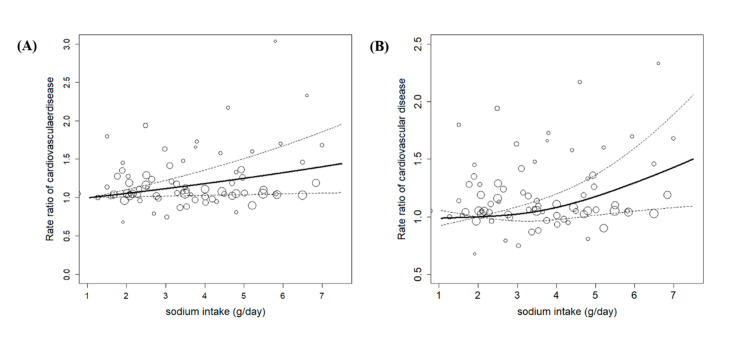
The linear (**A**) and non-linear (**B**) association of dietary sodium intake and cardiovascular disease. A, Heterogeneity: I2 = 72%; *p* ≤ 0.0001; B, Heterogeneity: I2 = 58%; *p* ≤ 0.0001.

Twenty studies which reported multiple categories of sodium intake were included in the dose-response meta-analysis (Figure 3). The RR in the linear model (Figure 3A) indicated that the risk of CVD significantly increased by 6% when 1 g dose of sodium was ingested (RR, 1.06; 95% CIs: 1.01–1.11). The Wald test indicated a significant difference between the two slopes in the nonlinear model (*p* = 0.04). The likelihood ratio test suggested linearity, and showed a linear relationship between dietary sodium intake and CVD risk.

### 3.4. Subgroup Analysis, Meta-Regression, and Sensitivity Analyses

The pooled RR in the 28 studies was significant (RR, 1.04; 95% CIs: 1.01–1.07), with evidence of heterogeneity (*p* < 0.0001, I2 = 77%, Figure S1). Subgroup analyses and meta-regression analyses for the dose-response and the coefficients between dietary sodium intake and CVD of eight studies are presented. Meta-regression showed that the risk of CVD due to an increment of 1 g per day was non-significant when modified by mean age, proportion of women, the duration of follow-up, percentage of white participants, body mass index and quality scores (Table 1 and Table 2). Sensitivity analyses showed that the overall results were not significantly affected by omitting any single study (Figure S2).

### 3.5. Publication Bias

In the 28 studies included in our analysis, 20 dose-response articles and eight studies reported the coefficients between dietary sodium intake and CVD, also, the funnel plot of the 28 studies showed no asymmetry, and the Egger’s test results presented no publication bias (*p* = 0.11; Figure S3). In the highest versus lowest meta-analysis, the Eggers’s test results indicated evidence of publication bias (*p* = 0.02; Figure S4).

## 4. Discussion

Our main findings demonstrate the relationship between high sodium intake and high CVD risk. We also found a significant linear relationship between dietary sodium intake and CVD risk. The risk of CVD increased up to 6% for every 1 g increase in sodium intake per day.

Our results are consistent with three previous meta-analyses in which high sodium intake was associated with the risk of CVD [15,19,70]. However, in another meta-analysis of 23 cohort studies, a U-shaped association between sodium intake and CVD was found. Participants with an intake of more than 215 mmol and less than 115 mmol had higher mortality compared to participants with a dietary sodium intake between 115–215 mmol [71]. The U-shaped association was found in several studies [69,72,73]. Nevertheless, in our meta-analysis, we did not find the U-shaped relationship between dietary sodium intake and CVD risk. Cobb and colleagues suggested that the inconsistencies in the relationship between sodium intake and CVD risk in different cohort studies are due to methodological errors, including reverse causality, systematic and random errors in sodium assessment and residual confounders [74]. The International Consortium for Quality Research on Dietary Sodium/Salt (INTERSALT) has provided some suggestions on the use of twenty-hour, spot, and short duration (<24 h) time urine collections to assess dietary sodium intake. To assess the accuracy of the 24 h urine collection, the use of para-aminobenzoic acid should also be considered. However, the estimation of para-aminobenzoic acid increases the cost of the studies and the burden on study subjects. Spot and short-term urine collection are more cost-effective compared to 24 h urine collection, however, the formula used to convert these to 24 h urine collection may result in an overestimation of the lower 24 h urine value and underestimation of the higher 24 h urine value [14]. Although the relationship between sodium intake and CVD risk was different among the studies, the premise that high sodium intake is related to high CVD risk has been demonstrated [19]. The results in the linear model were consistent with two previous dose-response meta-analyses [21,75]. Nevertheless, the World Health Organization recommends that daily sodium intake should be less than 2 g in adults [7]. A previous study showed that the mean global daily sodium intake was 3.95 g, approximately twice as much as the recommended 2 g, which is a threshold for CVD risk [76]. In 2010, a total of 1.65 million people died from CVD due to consuming more than 2 g of sodium per day [76]. We did not observe a threshold effect between daily sodium intake and CVD risk in our study and observed higher sodium intake with higher CVD risk in both highest versus lowest and dose-response meta-analysis. Higher sodium consumption is associated with a marked increase in the risk of CVD. Consuming a moderate amount of sodium should be encouraged in CVD prevention.

The pathophysiology of high CVD risk is considered to be consistent with high sodium intake, which decreases the function of the renin-angiotensin-aldosterone system and increases cardiac output [77,78,79,80]. Walker and colleagues evaluated 574 participants, and demonstrated that renin, angiotensin II and aldosterone had a significant negative correlation with BP [78]. Additionally, it has been reported that people with low-renin essential hypertension have increased extracellular and plasma fluid volumes. Twenty percent of patients with essential hypertension have suppressed plasma renin activity, which indicates a volume expanded state [81]. Another mechanism associated with high CVD risk is an increase in sodium intake, which increases the cardiac output and subsequently leads to increased BP. Higher cardiac output has two pathways that can lead to hypertension: the Starling mechanism and increasing peripheral resistance. Increased pressure leads to an increase in peripheral resistance, which is the main cause of the hypertension [79].

In our study, high degree of heterogeneity was evident after the meta-regression and subgroup analysis was conducted. Several investigators reported salt sensitivity, a concept of heterogeneity to explain the relationship between BP and variations in dietary sodium intake [80,82,83]. The definition of salt sensitivity in the normotensive individual is at least 3 mmHg reduction in mean arterial pressure following a period of dietary salt restriction [84]. The salt sensitivity among patients with hypertension was double compared with people with normal BP [84]. Additionally, age is also an important factor in salt sensitivity. In the INTERSALT study, a positive relationship between age differences and systolic BP was demonstrated [85]. Genetic factors serve as mediators for BP in salt sensitivity. People with haptoglobin 1-1 phenotype were more sensitive to salt than individuals with 2–1 or 2–2 phenotypes [86]. Salt-sensitive individuals were typically hypertensive. However, the relationship between salt sensitivity and CVD is not clear, which may be one of the reasons for the high heterogeneity in our study. Further studies are necessary to investigate the relationship between salt sensitivity and CVD outcomes.

Excess sodium intake was related to hypertension and CVD [15]. We suggest that the following three aspects should be considered to increase awareness of the benefits of low sodium intake in the general populations: knowledge, attitudes and practices. Land and colleagues reported that 95% of people are aware of the link between serious health problems and a high-salt diet. However, only 18% of the participants knew the recommended maximum dietary salt intake [87]. Although people realize that excess sodium intake is harmful to health, they may not understand how to reduce their intake of sodium. Policies related to sodium reduction and health education about low sodium diet are crucial for the general population to prevent CVD and other diseases.

Our study had several strengths. First, all of the included studies were cohort studies, with increased evidence of causal inference. Second, we used different meta-analysis methods to explore the relationship between sodium intake and CVD risk, and all the methods showed consistent results, namely, that high sodium intake is associated with a high risk of CVD. Third, we explored both linear and nonlinear relationship, which provide comprehensive information about the disadvantage of excess sodium intake.

However, our study has the following limitations. First, dietary questionnaires and urine collection methods might not accurately measure individual sodium intake. It is difficult to assess an individual’s dietary salt intake because it is difficult to determine the amount of salt that is added to food. Systematic errors remained and could not be corrected in the meta-analysis. Dietary assessment, including dietary records, 24 h dietary recalls and food frequency questionnaires were generally used to estimate individual nutrient intake. In the epidemiological studies, it was common to use food frequency questionnaires to assess long-term nutrient intake. Many researchers used 24 h dietary recall and dietary records to assess the validity of food frequency questionnaires. For sodium intake, the correlation coefficient between food frequency questionnaires and 7 day dietary records was 0.5, and the correlation coefficient between food frequency questionnaires and 24 h dietary recall was 0.2. Both recall and dietary records had measurement error, which leads to the underestimation of the validity of food frequency questionnaires [88]. Micheli et al. conducted a study to compare urine sodium collection with dietary records in sodium intake assessment. There was a weak inverse correlation between dietary records and 24 h urinary excretion for sodium intake assessment [89]. McLean et al. performed a meta-analysis to compare 24 h urine with 24 h dietary recall for dietary sodium intake assessment and found that using 24 h dietary recall to estimate sodium intake tended to underestimate dietary sodium intake. Complete 24 h urinary sodium collection was more reliable than dietary sodium assessment [90]. However, stratification by sodium assessment in the subgroup analysis showed a non-significant difference that was independent of whether urine collection or the diet questionnaires were used. Second, high heterogeneity remained even though we stratified the population by many potential confounders and conducted a meta-regression. More studies in the general population and more accurate tools of sodium assessment are required. We searched Embase and PubMed from their inception up to 17 August 2020, however, other studies may have been published after this date.

## 5. Conclusions

Our systematic review and meta-analysis provided evidence that high sodium intake is an important risk factor of CVD. The risk of CVD with high sodium intake compared to low sodium intake increased significantly by 19%. In the dose-response meta-analysis, we found a significant linear association between dietary sodium intake and risk of CVD. Every 1 g of dietary sodium intake increased the risk of CVD by 6%.

## Figures and Tables

**Table 1:** Summary of subgroup analysis for the association of dietary sodium intake and the risk of cardiovascular disease by subregion, follow-up years, sodium assessment and outcomes.

Subgroup Analysis	Number of Estimates	Summary RR (95% CI)	I^2^ (%) *	*p* Value *
Overall	33	1.04 (1.01, 1.07)	77	0.01 *
Subregion				0.06
Asia	9	1.15 (1.01, 1.29)	72	
Non-Asia	23	1.03 (0.99, 1.07)	75	
Multi-nation	1	1.00 (0.97, 1.02)	-	
Follow-up years				0.88
Above ten years	20	1.05 (1.00, 1.10)	67	
Less than 10 years	13	1.01 (1.01, 1.02)	85	
Sodium assessment				0.82
From diet	19	1.05 (0.99, 1.11)	76	
From Urine	14	1.04 (1.00, 1.08)	63	
Outcomes				0.91
Total CVD	14	1.05 (1.01, 1.09)	81	
CVD mortality	11	1.06 (0.94, 1.20)	81	
Stroke	5	1.04 (0.91, 1.19)	73	
Coronary heart disease	1	1.05 (0.97, 1.15)	-	
Heart failure	2	0.99 (0.89, 1.10)	62	

Abbreviations: RR, rate ratio; CI, confidence interval; CVD, cardiovascular disease. * I^2^ and *p* value related to subgroup differences.

**Table 2:** Summary of meta-regression for the association of dietary sodium intake and the risk of cardiovascular disease by mean age, percentage of women, follow up years, quality score of the study, body mass index and percentage of white participants.

Meta Regression	Number of Estimates	Point Estimates	I^2^ (%) *	*p* Value *
Mean age (year)	32	1.00 (0.99, 1.01)	90	0.92
Percentage of women (%)	33	1.00 (1.00, 1.00)	90	0.89
Follow-up years (year)	33	1.00 (0.99, 1.01)	90	0.89
Study quality score	33	1.01 (0.96, 1.06)	86	0.60
Body mass index (kg/m^2^)	22	0.99 (0.97, 1.02)	91	0.62
Percentage of white participants (%)	10	1.00 (1.00, 1.01)	83	0.75

* I^2^ and *p* value related to meta regression analysis.

## Supplementary Materials

The following are available online at https://www.mdpi.com/2072-6643/12/10/2934/s1, Supplementary S1: PRISMA checklist Study., Supplementary S2: Protocol and Search Strategies., Figure S1: Forest plot for the coefficients of dietary sodium intake and risk of total cardiovascular disease in 28 studies., Figure S2: Sensitivity analyses of dietary sodium intake and risk of total cardiovascular disease by omitting each study., Figure S3: Funnel plot of total cardiovascular disease for coefficients of dietary sodium intake in 28 studies of β-coefficients type., Figure S4: Funnel plot of total cardiovascular disease for highest versus lowest categories of dietary sodium intake in 24 studies of highest versus lowest type., Table S1:Characteristics of the studies included in the systematic review, Table S2: Newcastle−Ottawa scale for assessment of quality of included cohort studies.
