# Supplementary material for: Dietary Sodium Intake and Risk of Cardiovascular Disease: A Systematic Review and Dose-Response Meta-Analysis
Source: Nutrients. 2020 Sep 25;12(10):2934. doi: 10.3390/nu12102934 (PMC7601012; doi:10.3390/nu12102934)
Supplement: Supplementary file 1 [file nutrients-12-02934-s001.pdf]

Supplemental data

Wang YJ, Yeh TL, Shih MC, et al. Dietary Sodium Intake and The Risk of Cardiovascular Disease : A Systematic Review and Dose-Response Meta-analysis

Supplementary S1. PRISMA checklist Study
Supplementary S2. Protocol and Search Strategies
Figure S1. Forest plot for the coefficients of dietary sodium intake and risk of total cardiovascular disease in 28 studies.
Figure S2. Sensitivity analyses of dietary sodium intake and risk of cardiovascular disease by omitting each study.
Figure S3. Funnel plot of cardiovascular disease for coefficients of dietary sodium intake in 28 studies of β-coefficients type.
Figure S4. Funnel plot of cardiovascular disease for highest versus lowest categories of dietary sodium intake in 24 studies of highest versus lowest type .
Table S1. Characteristics of the studies included in the systematic review.
Table S2. Newcastle–Ottawa scale for assessment of quality of included cohort studies.

Supplemental References. References for Table S1 and Table S2.

Supplementary S1. PRISMA checklist.

Section/topic	#	Checklist item	Reported on page #
**TITLE**			
Title	1	Identify the report as a systematic review, meta-analysis, or both.	1
ABSTRACT			
Structured summary	2	Provide a structured summary including, as applicable: background; objectives; data sources; study eligibility criteria, participants, and interventions; study appraisal and synthesis methods; results; limitations; conclusions and implications of key findings; systematic review registration number.	1
INTRODUCTION			
Rationale	3	Describe the rationale for the review in the context of what is already known.	1-2
Objectives	4	Provide an explicit statement of questions being addressed with reference to participants, interventions, comparisons, outcomes, and study design (PICOS).	2
METHODS			
Protocol and registration	5	Indicate if a review protocol exists, if and where it can be accessed (e.g., Web address), and, if available, provide registration information including registration number.	2
Eligibility criteria	6	Specify study characteristics (e.g., PICOS, length of follow-up) and report characteristics (e.g., years considered, language, publication status) used as criteria for eligibility, giving rationale.	2
Information sources	7	Describe all information sources (e.g., databases with dates of coverage, contact with study authors to identify additional studies) in the search and date last searched.	2
Search	8	Present full electronic search strategy for at least one database, including any limits used, such that it could be repeated.	2-3
Study selection	9	State the process for selecting studies (i.e., screening, eligibility, included in systematic review,	2-3

		and, if applicable, included in the meta-analysis).	
Data collection process	10	Describe method of data extraction from reports (e.g., piloted forms, independently, in duplicate) and any processes for obtaining and confirming data from investigators.	3
Data items	11	List and define all variables for which data were sought (e.g., PICOS, funding sources) and any assumptions and simplifications made.	3
Risk of bias in individual studies	12	Describe methods used for assessing risk of bias of individual studies (including specification of whether this was done at the study or outcome level), and how this information is to be used in any data synthesis.	3
Summary measures	13	State the principal summary measures (e.g., risk ratio, difference in means).	3
Synthesis of results	14	Describe the methods of handling data and combining results of studies, if done, including measures of consistency (e.g., I2) for each meta-analysis.	3-4

Section/topic	#	Checklist item	Reported on page #
Risk of bias across studies	15	Specify any assessment of risk of bias that may affect the cumulative evidence (e.g., publication bias, selective reporting within studies).	3-4
Additional analyses	16	Describe methods of additional analyses (e.g., sensitivity or subgroup analyses, meta-regression), if done, indicating which were pre-specified.	3-4
RESULTS			
Study selection	17	Give numbers of studies screened, assessed for eligibility, and included in the review, with reasons for exclusions at each stage, ideally with a flow diagram.	4-5,Figure 1
Study characteristics	18	For each study, present characteristics for which data were extracted (e.g., study size, PICOS, follow-up period) and provide the citations.	4,
Risk of bias within	19	Present data on risk of bias of each study and, if available, any outcome level assessment	4, Table S2

studies		(see item 12).	
Results of individual studies	20	For all outcomes considered (benefits or harms), present, for each study: (a) simple summary data for each intervention group (b) effect estimates and confidence intervals, ideally with a forest plot.	4-8, Table S1, Figure 2, Figure 3
Synthesis of results	21	Present results of each meta-analysis done, including confidence intervals and measures of consistency.	4
Risk of bias across studies	22	Present results of any assessment of risk of bias across studies (see Item 15).	4
Additional analysis	23	Give results of additional analyses, if done (e.g., sensitivity or subgroup analyses, meta-regression [see Item 16]).	7-8,Table 1,Table 2
DISCUSSION			
Summary of evidence	24	Summarize the main findings including the strength of evidence for each main outcome; consider their relevance to key groups (e.g., healthcare providers, users, and policy makers).	8
Limitations	25	Discuss limitations at study and outcome level (e.g., risk of bias), and at review-level (e.g., incomplete retrieval of identified research, reporting bias).	9-10
Conclusions	26	Provide a general interpretation of the results in the context of other evidence, and implications for future research.	10
FUNDING			
Funding	27	Describe sources of funding for the systematic review and other support (e.g., supply of data); role of funders for the systematic review.	8

Supplementary S2. Search Strategies

Search Strategies

We will search the following electronic databases:

1. PubMed
2. Embase

There will be a restriction on language of publication in English.

We will search additional studies in the reference lists of all identified publications, including relevant meta-analyses and systematic reviews.

1. PubMed:

Search using the NCBI interface from the earliest available date of indexing through August 17, 2020

("Cardiovascular Diseases"[Mesh]) AND "Sodium, Dietary"[Mesh]) OR "Sodium Chloride, Dietary"[Mesh]) AND "Cohort Studies"[Mesh] OR "urinary sodium excretion"AND"humans"

2. Embase:

Search using the Elsevier interface from the earliest available date of indexing through August 17, 2020

('sodium intake'/exp OR 'dietary sodium' OR 'intake, sodium' OR 'natrium intake' OR 'sodium intake' OR 'sodium, dietary' OR 'sodium urine level'/exp OR 'diuresis, saline' OR 'diuresis, sodium' OR 'renal sodium excretion' OR 'sodium diuresis' OR 'sodium excretion, urinary' OR 'sodium loss' OR 'sodium urine level' OR 'urinary salt' OR 'urinary sodium' OR 'urinary sodium excretion' OR 'urine salt' OR 'urine sodium' OR 'salt intake'/exp OR 'dietary salt' OR 'dietary sodium chloride' OR 'intake, salt' OR 'salt intake' OR 'sodium chloride intake' OR 'sodium chloride, dietary') AND ('cardiovascular disease'/exp OR 'angiocardiopathy' OR 'angiocardiovascular disease' OR 'cardiovascular complication' OR 'cardiovascular disease' OR 'cardiovascular diseases' OR 'cardiovascular disorder' OR 'cardiovascular disturbance' OR 'cardiovascular lesion' OR 'cardiovascular syndrome' OR 'cardiovascular vegetative disorder' OR 'complication, cardiovascular' OR 'disease, cardiovascular' OR 'major adverse cardiovascular event') AND ('cohort analysis'/exp OR 'analysis, cohort' OR 'cohort analysis' OR 'cohort fertility' OR 'cohort life cycle' OR 'cohort studies' OR 'cohort study' OR 'fertility, cohort') AND [humans]

Figure S1. Forest plot for the coefficients of dietary sodium intake and risk of total cardiovascular disease[a] in 28 studies.

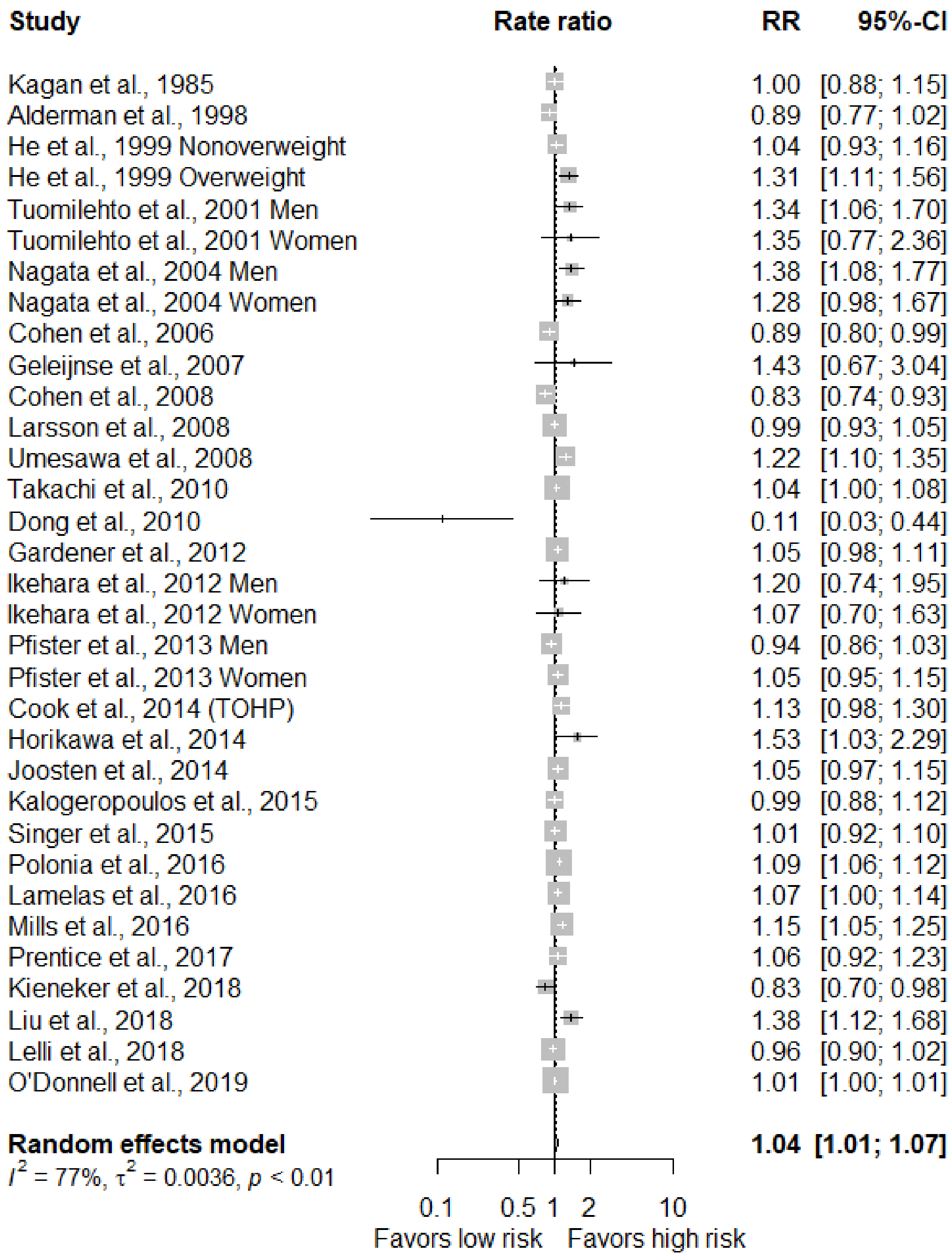

a:Total cardiovascular disease:The total cardiovascular disease included total CVD, CVD mortality, stroke, coronary heart disease, myocardial infarction and heart failure.

Figure S2. Sensitivity analyses of dietary sodium intake and risk of total cardiovascular disease[a] by omitting each study.

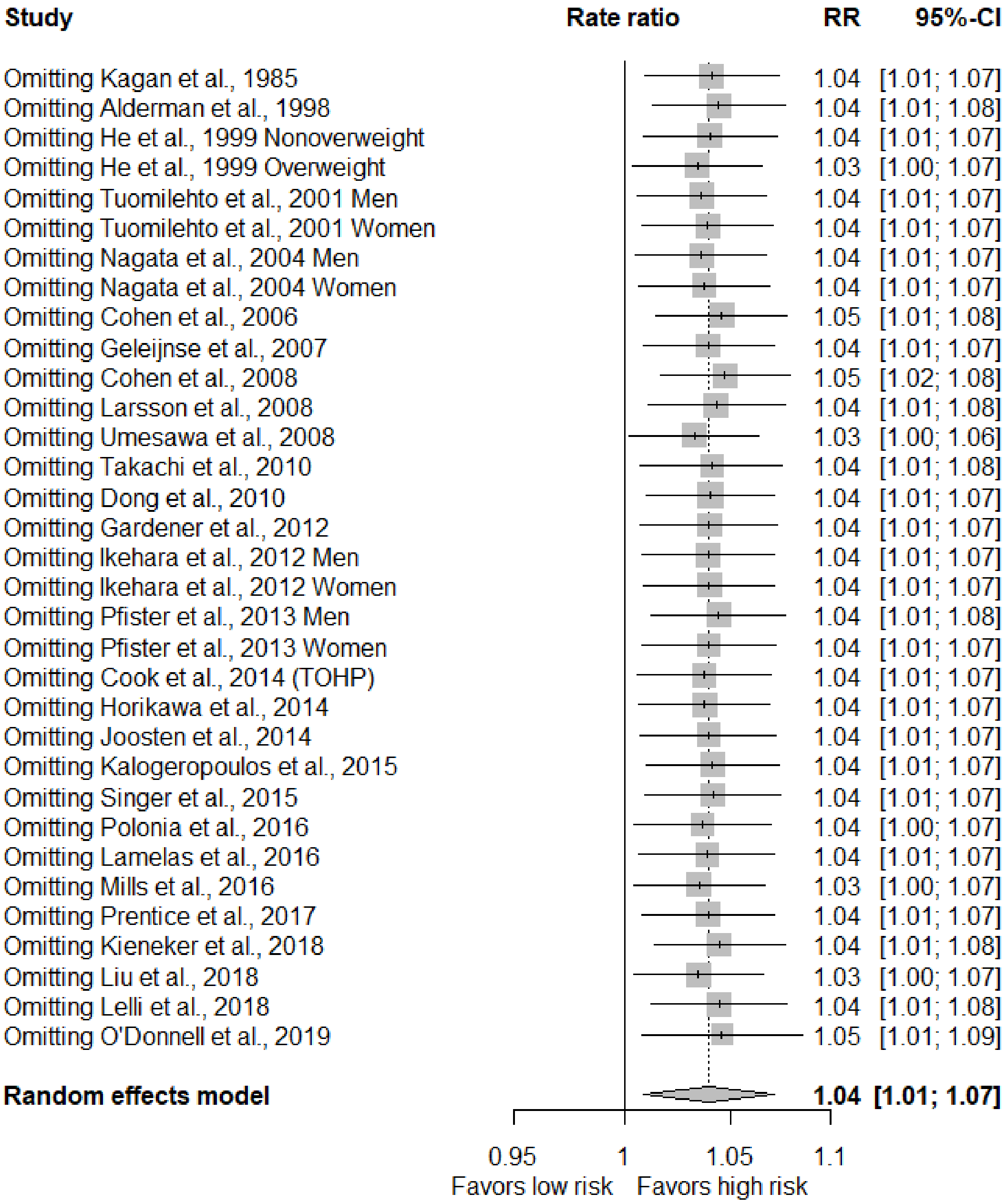

a:Total cardiovascular disease:The total cardiovascular disease included total CVD, CVD mortality, stroke, coronary heart disease, myocardial infarction and heart failure.

Figure S3. Funnel plot of total cardiovascular disease[a] for coefficients of dietary sodium intake in 28 studies of β-coefficients type.

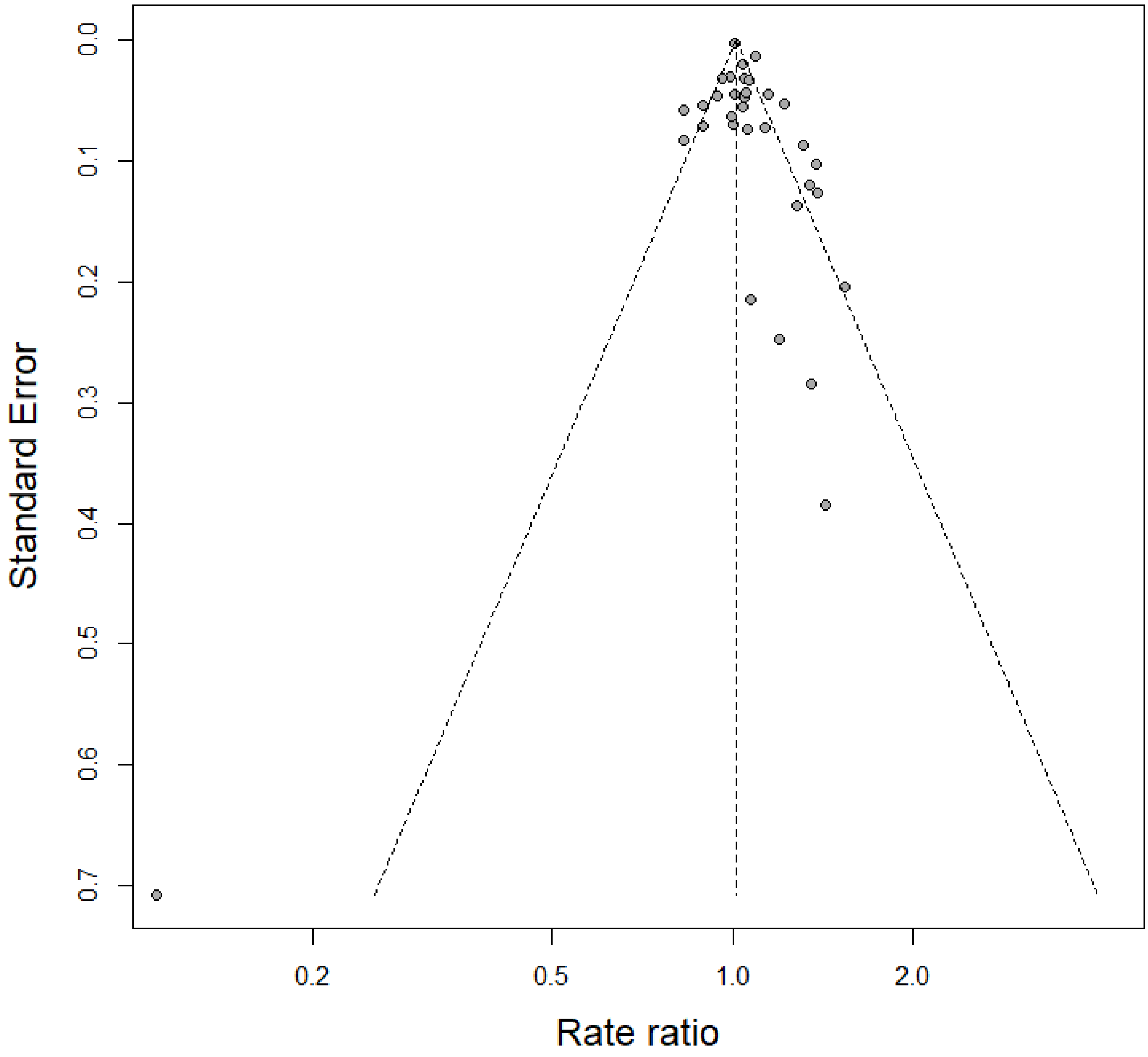

Egger test, slope= 0.01, p = 0.11

a:Total cardiovascular disease:The total cardiovascular disease included total CVD, CVD mortality, stroke, coronary heart disease, myocardial infarction and heart failure.

Figure S4. Funnel plot of total cardiovascular disease for highest versus lowest categories of dietary sodium intake in 24 studies of highest versus lowest type.

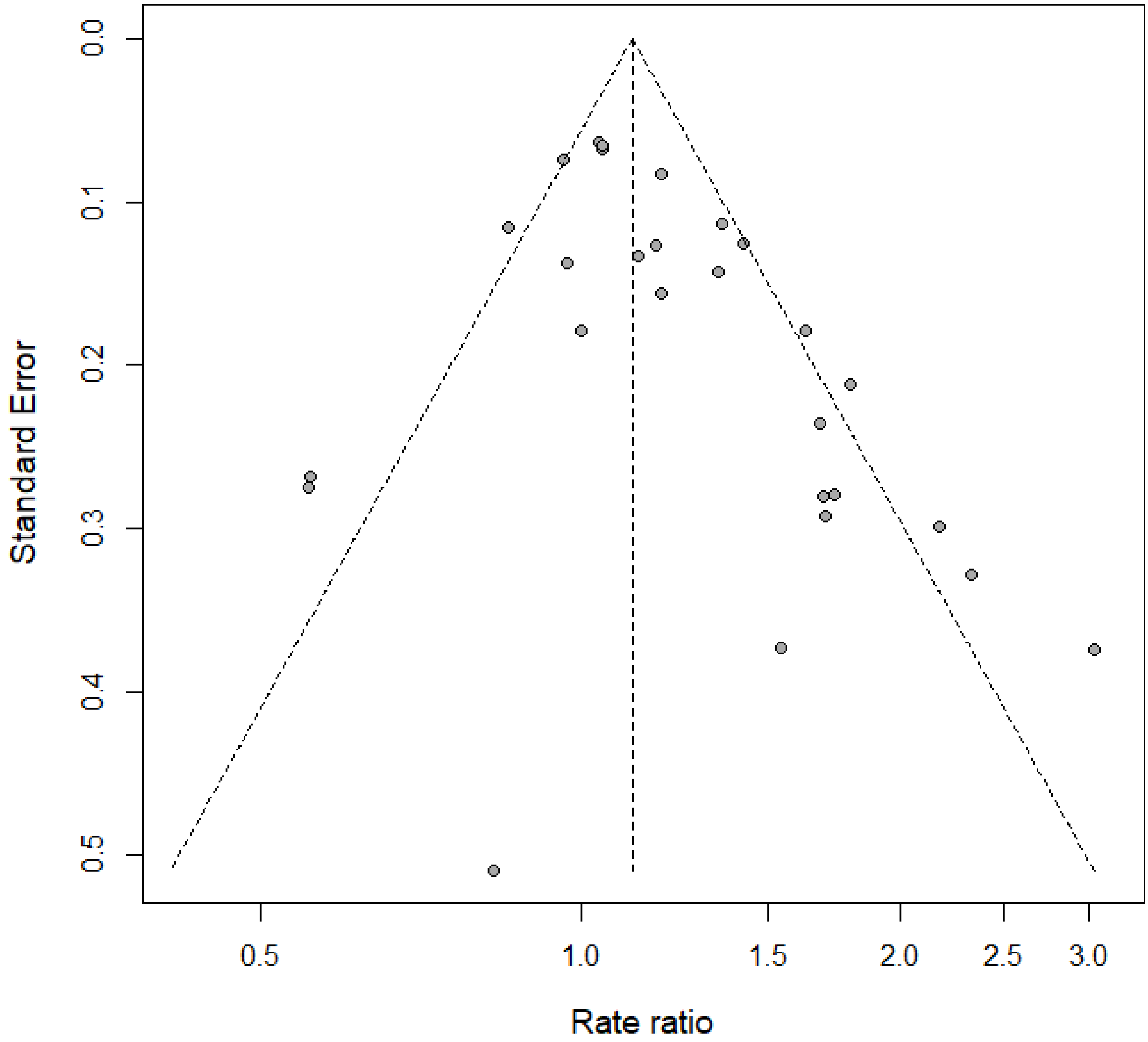

Egger test, slope= -0.05, p = 0.02

Table S1. Characteristics of the studies included in the systematic review.

Study	Region, follow-up duration, mean age and women proportion of participants	No. of case/total of person-years (PY) or numbers (N)	Sodium intake (24-hour) and assessment	Adjusted variables	Outcomes, presented with rate ratios and 95% confidence intervals
Kagan et al., 1985[1]	Hawaii, 10yrs f/u, 56.9 yrs, 0% W	238/7895 (n/N)	24-hour dietary recall; Q1, Q2, Q3, Q4, Q5: ≤ 1.78/1.79, 2.39/ 2.40, 3.00 3.01, 3.86/≥ 3.87 (g)	Age	Stroke; Q1, Q2, Q3, Q4, Q5:1.00 (reference), 1.05 (0.79, 1.44), 0.79 (0.37, 1.21), 1.07 (0.68, 1.46), 0.95 (0.55, 1.35)
Hu et al., 1992[2]	Taiwan, 4 yrs f/u, not reported yrs, not reported % W	No salty food (n/PY): 71/25082, Yes salty food (n/PY): 33/6420	Household survey questionnaire; yes/no salty food	Age	Stroke; 1.79 (1.18, 2.71)
Alderman et al., 1998[3]	USA,17-21 yrs f/u, 49.8 yrs, 60.5 % W	1970/11346 (n/N)	24-hour dietary recall; W: Q1, Q2, Q3, Q4: 678, 1232, 1791, 3105 (mg); M: Q1, Q2, Q3, Q4: 1041, 1832, 2647, 4538 (mg)	Age, sex, race, BMI, history of CVD, HTN, SBP	CVD mortality; 0.89 (0.77, 1.02)
He et al., 1999[4]	USA, 19yrs f/u, non-overweight: Q1, Q2, Q3, Q4: 46.2, 48.3, 49.3, 48.6 yrs; 62.1, 60.5, 57.3, 58.0% W; overweight: Q1,	Non-overweight (n/PY): Q1, Q2, Q3, Q4: 95/2825, 116/28853, 110/28666, 109/27698; overweight (n/PY): Q1, Q2, Q3, Q4: 45/11920, 61/10644, 75/10037, 69/11188	24-hour dietary recall; Non-overweight: Q1, Q2, Q3, Q4: 50.5, 76.9, 99.1, 142.5 (mmol); overweight: 45.5, 69.4, 88.7, 129.7 (mmol)	Age, sex, race, BMI, PA, education, drinking, smoking, energy intake, SBP,TC, history of DM, diuretic use	Stroke; non-overweight: Q1, Q2, Q3, Q4:1.00 (reference)/ 1.28 (0.99, 1.67), 1.04 (0.83, 1.31), 1.18 (0.92, 1.51); overweight: Q1, Q2, Q3, Q4:1.00 (reference),1.01 (0.68, 1.49), 1.28 (0.89, 1.85), 1.63 (1.16, 2.27)

	Q2, Q3, Q4: 50.0, 51.1, 52.0, 51.3 yrs; 64.1, 66.5, 63.5, 67.3% W				
Tuomileh to et al., 2001[5]	Finland,8-13 yrs f/u, W: 45.4 yrs, M: 45yrs, 51.8 % W	M (n/N): 72/1173; W (n/N): 87/1263	24-hour urinary sodium excretion; W: 154 (mmol); M: 204 (mmol)	Age, study year, BMI, smoking, HDL-C, SBP	CAD; M:1.34 (1.06, 1.70); W: 1.35 (0.77, 2.35)
He et al., 2002[6]	USA,19 yrs f/u, non-overweight: 48.2 yrs; 64% W; overweight: 52.2 yrs, 56% W	Nonoverweight (n/N): Q1, Q2, Q3, Q4:110/1190, 125/1131, 91/1351, 87/1381; overweight (n/N): Q1, Q2, Q3, Q4: 208/1401, 177/1279, 146/1239, 148/1210	24-hour urinary sodium excretion; non-overweight: 35.2 , 63, 92.7, 164.2 (mmol); overweight: 33.7, 62.9, 92.8, 167.6 (mmol)	Age, sex, race, energy intake, education, PA, smoking, drinking, history of DM and VHD, SBP, TC, time-dependent history of CAD, K and Ca intake	Congestive heart failure; non-overweight: Q1, Q2, Q3, Q4: 1.00 (reference), 1.04 (0.81, 1.33), 0.93 (0.69, 1.26), 0.87 (0.58, 1.31); overweight: Q1, Q2, Q3, Q4: 1.00 (reference), 1.09 (0.86, 1.37), 1.05 (0.78, 1.42), 1.43 (1.07, 1.91)
Nagata et al., 2004[7]	Japan, 7 yrs f/u, W: 53.3, 54.3, 57.8 yrs; M: Q1,Q2 Q3: 51.0, 53.2, 57.7 yrs, 54.3% W	M (n/PY): Q1, Q2, Q3: 23/30670, 40/30779, 74/29587 (PY); W (n/PY): Q1, Q2, Q3: 40/36719, 39/36874, 53/6530	Food frequency questionnaire; W: Q1, Q2, Q3: 3799 4801, 5930 (mg); M: Q1, Q2, Q3: 4070, 5209, 6613 (mg)	Age, energy intake, marital status, education, BMI, smoking, drinking, PA, histories of HTN and DM, protein, K and vitamin E	Total stroke death; M: Q1, Q2, Q3: 1.00 (reference), 1.60 (0.92, 2.80), 2.33 (1.23, 4.45); W: Q1, Q2, Q3: 1.00 (reference), 1.33 (0.80, 2.21), 1.70 (0.96, 3.02)
Cohen et al., 2006[8]	USA, 13.7 yrs f/u, 48 yrs, 53 % W	541/7154 (n/N)	24-hour dietary recall; 2718 (mg)	Age, sex, race, smoking, drinking, SBP, anti-hypertensive treatment, BMI, education, PA, BMI, K and energy intake, history of DM, TC	Total CVD; 0.89 (0.80, 0.99)

Cook et al., 2007[9]	USA, TOHP I: 10 yrs f/u, TOHP II: 15yrs f/u, 43.3 yrs, 31.2 % W	TOHP I (n/N) I, C:17/231, 71/938; TOHP II (n/N) I, C:32/311, 80/935	24-hour urinary sodium excretion; TOHP I: I, C: 154.6, 156.4 (mmol); TOHP II: I, C: 182.9, 184.5 (mmol)	Age, race, sex, weight loss intervention, baseline weight, sodium excretion	Total CVD; TOHP I: 0.48 (0.25, 0.92); TOHP II: 0.79 (0.57, 1.09)
Geleijnse et al., 2007[10]	Netherlands, 5.5 yrs f/u, 69.2 yrs, 59 % W	217/1448 (n/N)	24-hour urinary sodium excretion; 117 (mmol)	Age, sex, BMI, smoking, drinking, education DM, diuretics use, energy, Ca and saturated fat intake, urine Cr	CVD mortality; 1.43 (0.67, 3.03)
Cohen et al., 2008[11]	USA, 8.7 yrs f/u, Q1, Q2, Q3, Q4: 51, 49, 48, 44 yrs; 76.2, 62.1, 50.2, 31.9% W	Q1, Q2, Q3, Q4 (n/N): 159/2174, 126/2175, 103/2175, 48/2175	24-hour dietary recall; Q1, Q2, Q3, Q4: 1501, 2483, 3441, 5497 (mg)	Age, sex, race, education, added table salt, PA, drinking, smoking, history of DM, cancer, SBP, TC, K intake, weight, treatment for HTN	Total CVD; Q1, Q2, Q3, Q4: 1.80 (1.05, 3.08), 1.94 (1.32, 2.85), 1.48 (0.82, 2.67), 1.00 (reference)
Larsson et al., 2008[12]	Sweden, 13.6 yrs f/u, 50-69 yrs, 0% W	2702/360187 (n/PY)	Food frequency questionnaire; Q1, Q2, Q3, Q4, Q5: 3909, 4438, 4810, 5212, 5848 (mg)	Age, supplementation group, smoking, drinking, BMI, SBP, DBP, TC, HDL-C, histories of DM, CHD, PA, energy intake	Cerebral infarction; Q1, Q2, Q3, Q4, Q5: 1.00 (reference), 1.08 (0.96, 1.22), 1.05 (0.93, 1.18), 0.99 (0.87, 1.13), 1.04 (0.92, 1.18)
Umesawa et al., 2008[13]	Japan, 12.7 yrs f/u, Q1, Q2, Q3, Q4, Q5: 55, 56, 56, 56, 58 yrs; 45, 64, 63,64, 67% W	Q1, Q2, Q3, Q4,Q5 (n/N): 338/11746, 344/11746,402/11746, 463/11746, 540/11746	Food frequency questionnaire; Q1, Q2, Q3, Q4,Q5: 50, 73, 90, 109, 135 (mmol)	Age, sex, BMI, smoking, drinking, history of HTN, DM, menopause, HRT, PA, walking time, education, mental stress, Ca and K intake	CVD mortality; Q1, Q2, Q3, Q4,Q5 :1.00 (reference), 1.04 (0.89, 1.22), 1.19 (1.01, 1.39), 1.29 (1.10, 1.52), 1.42 (1.20, 1.69)

Dong et al ., 2010[14]	China, 31.4 months f/u, 59.4 yrs, 57.7% W	32/305 (n/N)	3-day dietary records; 1.82 (g)	Age, sex, BMI, DM, history of CVD, MAP, Hb, Alb, Ca , P, LDL-C, Kt/V, CCr	CVD mortality; 0.11 (0.03, 0.48)
Takachi et al., 2010[15]	Japan, 6-9 yrs f/u, Q1, Q2, Q3, Q4, Q5: 56.1, 56.4, 56.7, 57.1, 57.9 yrs; 38.3, 48.1, 54.0, 61.1, 68.1% W	Q1, Q2, Q3, Q4,Q5 (n/N): 416/15500, 428/15500, 386/15500, 403/15500, 433/15500	Food frequency questionnaire; Q1, Q2, Q3, Q4,Q5: 3084, 4005, 4709, 5503, 6844 (mg)	Age, sex, BMI, smoking, drinking, PA, energy, K and Ca intake	Total CVD; Q1, Q2, Q3, Q4,Q5: 1.00 (reference), 1.11 (0.96, 1.29), 1.02 (0.87, 1.19), 1.10 (0.94, 1.29), 1.19 (1.01, 1.40)
Yang et al., 2011[16]	USA, 14.8 yrs f/u, not reported yrs, 52% W	825/170110 (n/PY)	24-hour dietary recall; Low: 2176 (mg); high: 5135 (mg)	Sex, race, education, BMI, smoking, drinking, TC, HDL-C, PA, family history of CVD, energy intake	CVD mortality; 0.83 (0.31, 2.28)
Gardener et al., 2012[17]	USA, 10 yrs f/u, Q1, Q2, Q3, Q4: 69, 70, 69, 68 yrs; 64, 79, 65, 51% W	Q1, Q2, Q3, Q4 (n/N): 67/3306, 157/6432, 253/11447, 138/5095	Food frequency questionnaire; Q1, Q2, Q3, Q4, Q5: 65.2, 82.6, 137.0, 304.3 (mmol)	Age, sex, race, education, smoking, drinking, PA, energy, protein, fat, saturated fat and carbohydrates intake, DM, hypercholesterolemia, HTN, history of CVD, BMI	Stroke, myocardial infarction or CVD mortality; Q1, Q2, Q3, Q4: 1.00 (reference), 1.35 (1.00, 1.82), 1.21 (0.87, 1.67), 1.68 (1.06, 2.67)
Ikehara et al., 2012[18]	Japan, 16.4yrs f/u, W: Q1, Q2, Q3: 58.1, 57.3, 56.9 yrs; M: Q1, Q2, Q3: 58.5, 57.9, 55.7 yrs, 58.1% W	M: Q1, Q2, Q3 (n/PY): 306/59903, 1117/230090, 895/214563; W: Q1, Q2, Q3 (n/PY): 415/131986, 1108/399263, 576/188299	Self-administered questionnaire; W: Q1, Q2, Q3 (mg): 1725, 1950, 2022; M: Q1, Q2, Q3: 1878, 2083, 2051 (mg)	Age, BMI, history of DM, HTN, smoking, drinking, education, PA, walking, mental stress, fresh fish intake	CVD mortality; M: Q1, Q2, Q3: 1.00 (reference), 1.02 (0.90, 1.16), 1.05 (0.92, 1.20) W: Q1, Q2, Q3: 1.00 (reference), 0.96 (0.86, 1.08), 1.05 (0.92, 1.19)

Cook et al., 2014[19]	TOHP I: USA, 10 yrs f/u, W: Q1, Q2, Q3, Q4: 44.6, 44.7, 43.0, 42.7 yrs; M: Q1, Q2, Q3, Q4: 42.1, 42.8, 43.3, 42.7 yrs, 29% W<br>TOHP II: USA, 15 yrs f/u, W: Q1, Q2, Q3, Q4: 44.4, 43.8, 43.1, 44.0 yr; M: Q1, Q2, Q3, Q4: 42.7, 43.6, 43.6, 42.5 yrs, 32.9% W	TOHP I:Q1, Q2, Q3 (n/N): 15/1890, 48/5900, 40/4270, 23/1910<br>TOHP II: Q1, Q2, Q3 (n/N): 2/705, 13/4545, 34/5115, 18/3360	TOHP I:24-hour urinary sodium excretion; W: Q1, Q2, Q3, Q4,Q5:1847.6, 2908.1, 4088.6, 5941.2 (mg); M: Q1, Q2, Q3, Q4: 1928.2, 3054.5, 4145.8, 5923.0 (mg)<br>TOHP II: 24-hour urinary sodium excretion; W: Q1, Q2, Q3, Q4:1965.0, 3032.8, 4034.6, 5596.7 (mg); M: Q1, Q2, Q3, Q4:1995.2 3119.5, 4200.5, 5728.8 (mg)	TOHP I:Age, sex, race, clinic, treatment assignment, education, baseline weight, drinking, smoking, PA, urine K, family history of CVD, weight changes, smoking, PA during the trial periods<br>TOHP II: Age, sex, race, clinic, treatment assignment, education, baseline weight, drinking, smoking, PA, urine K, family history of CVD, weight changes, PA, during the trial periods	TOHP I:Total CVD; Q1, Q2, Q3, Q4: 0.68 (0.34, 1.37), 0.75 (0.50, 1.11), 1.00 (reference), 1.05 (0.68, 1.62)<br>TOHP II: Total CVD; Q1, Q2, Q3, Q4: 0.68 (0.34, 1.37), 0.75 (0.50, 1.11), 1.00 (reference), 1.05 (0.68, 1.62)
Horikawa et al., 2014[20]	Japan, 8yrs f/u, Q1, Q2, Q3, Q4: 58.1, 58.6, 59.0, 59.1 yrs; 49.6, 51.4, 47.2, 42.0% W	Q1, Q2, Q3, Q4 (n/N):23/354, 36/350, 32/351, 41/359	Food frequency questionnaire; Q1, Q2, Q3, Q4: 3.3, 3.8, 4.4, 4.6 (g)	Age, sex, BMI, HbA1c, DM duration, LDL-C, HDL-C, TG, insulin, lipid-lowering agents, smoking, drinking, energy intake, PA, SBP, antihypertensive agents	Total CVD; Q1, Q2, Q3, Q4: 1.00 (reference), 1.73 (1.00, 3.00), 1.58 (0.88, 2.84), 2.17 (1.21, 3.90)
Joosten et al., 2014[21]	Netherlands, 10.5yrs f/u, Q1, Q2,	Q1, Q2, Q3, Q4 (n/PY): 123/17638, 111/17975, 112/17878, 106/18000	24-hour urinary sodium excretion; W : Q1, Q2, Q3, Q4: <95, 95, 121, 122,	Age, sex, BMI, smoking, drinking, family history of	CHD; Q1, Q2, Q3, Q4: 1.00 (reference), 0.99 (0.76, 1.29), 1.09 (0.83, 1.44), 1.19 (0.88, 1.62)

	Q3, Q4: 50, 49, 48, 47 yrs, 51.3% W		151, >151 (mg); M: Q1, Q2, Q3, Q4: <122, 122, 154,155, 190, >190 (mg)	CHD, DM, TC/HDL-C, urine Mg, K, Cr	
O'donnell et al., 2014[22]	17 countries[a], 3.7 yrs f/u, 51 yrs, 57.5% W	Q1, Q2, Q3, Q4, Q5 (n/N): 462/10810, 662/21131, 1437/46663, 391/12324, 365/11017	24-hour urinary sodium excretion; Q1, Q2, Q3, Q4, Q5: 2.44, 3.54, 4.93, 6.45, 8.31 (g)	Age, sex, education, smoking, drinking, DM, BMI, PA, use of CVD agents, history of CVD, TB, cancer, HIV	Death or total CVD; Q1, Q2, Q3, Q4, Q5:1.24 (1.07, 1.42), 1.00 (0.91, 1.10), 1.00 (reference), 1.06 (0.95, 1.19), 1.14 (1.01, 1.29)
Pfister et al., 2014[23]	United Kingdom, 12.9yrs f/u, W: Q1, Q2, Q3, Q4, Q5: 58.9, 57.5, 57.2, 57.2, 67.8 yrs; M: Q1, Q2, Q3, Q4, Q5: 59.4, 58.5, 58.2, 57.5, 58.1 yrs, 54.6% W	M: Q1, Q2, Q3, Q4, Q5 (n/N): 167/1803, 131/1803, 127/1804, 127/1803, 150/1804; W: Q1, Q2, Q3, Q4, Q5 (n/N): 112/2168, 92/2168, 82/2168, 90/2168, 132/2168	Single spot urine sodium excretion; W: Q1, Q2, Q3, Q4, Q5:101, 133, 154, 175, 216 (mmol); M: Q1, Q2, Q3, Q4, Q5: 115, 145, 163, 182, 218 (mmol)	Age, BMI, DM, TC, social class, education, smoking, drinking, PA, BP	Heart failure; M: Q1, Q2, Q3, Q4, Q5: 1.24 (0.98, 1.56), 1.00 (reference), 0.97 (0.76, 1.24), 0.98 (0.77, 1.26), 1.06 (0.84, 1.35); W: Q1, Q2, Q3, Q4, Q5: 1.11 (0.84, 1.46), 1.00 (reference), 0.88 (0.65, 1.18), 0.93 (0.70, 1.25), 1.26 (0.96, 1.65)
Kalogero pouloset al., 2015[24]	USA, 10 yrs f/u, 73.6 yrs, 51.2% W	Q1, Q2, Q3 (n/PY): 63/ 217, 161/ 576, 348/1188	Food frequency questionnaire;Q1, Q2, Q3: <1500, 1500, 2300, >2300 (mg)	Age, sex, race, HTN, BMI, smoking, PA, history of CVD, pulmonary disease, DM, depression, BP, HR, EKG, serum glucose, Alb, Cr, TC	Total CVD; Q1, Q2, Q3: 1.05 (0.79, 1.41), 1.00 (reference), 1.02 (0.84, 1.24)
Singer et al., 2015[25]	USA, 6.5yrs f/u, Q1, Q2, Q3, Q4: 53.7, 53.1, 51.5, 51.0 yrs;	Q1, Q2, Q3, Q4 (n/N): 128/890, 97/876, 96/865, 78/874	24-hour urinary sodium excretion; Q1, Q2, Q3,	Age, sex, race, BMI, SBP, eGFR, urine K, hematocrit, PRA,	CVD mortality; Q1, Q2, Q3, Q4: 1.00 (0.71, 1.42), 0.96 (0.68, 1.36), 1.06 (0.75, 1.49), 1.00 (reference)

	36.6, 35.3, 36.2, 36.0% W		Q4: 55, 102, 143, 221 (mmol)	DM, smoking, history of baseline LVH	
Lamelas et al., 2016[26]	USA, 4.7 yrs f/u, 51.5 yrs, 59.7 % W	Q1, Q2, Q3, Q4, Q5, Q6 (n/N): 50/1638, 115/3885, 161/4758, 110/3457, 73/1748, 59/1063	24-hour urinary sodium excretion; Q1, Q2, Q3, Q4, Q5, Q6: <3, 3, 3.99, 4, 4.99, 5, 5.99, 6, 6.99, >7 (g)	Age, sex, BMI, smoking, drinking, DM, education, history of CVD, country, LDL-C/HDL-C, energy, fruit and vegetable intake, SBP, history of HTN, antihypertensive agents	Total CVD; Q1, Q2, Q3, Q4, Q5, Q6: 1.14 (0.73, 1.78), 1.14 (0.82, 1.59), 1.05 (0.77, 1.43), 1.00 (reference), 1.46 (0.95, 2.26), 1.93 (1.20, 3.09)
Mills et al., 2016[27]	USA, 6.8 yrs f/u, Q1, Q2, Q3, Q4: 57.2, 57.7, 58.2, 58.0 yrs; 65.0, 50.1, 38.7, 24.0% W	Q1, Q2, Q3, Q4 (n/PY): 174/5804, 159/5972, 198/5739, 273/5012	24-hour urinary sodium excretion; Q1, Q2, Q3, Q4: 2491, 3364, 4008, 4941 (mg)	Age, sex, race, clinic site, education, WC, lean BMI, BMI, smoking, drinking, PA; LDL-C, glucose, history of CVD, use of antidiabetic agents, lipid-lowering agents, diuretics, RAS blockades, antihypertensive agents, urinary Cr excretion, eGFR	Total CVD; Q1, Q2, Q3, Q4: 1.00 (reference), 0.87 (0.69, 1.10), 1.01 (0.81, 1.26), 1.36 (1.09, 1.70)
Polonia et al., 2016[28]	Portugal, 7.2 yrs f/u, 54.1 yrs, 56.3% W	122/608 (n/N)	24-hour urinary sodium excretion; Low, high: 198, 260 (mmol)	Age, night-time SBP	Total CVD; 1.09 (1.06, 1.12)
Olde Engberin	Neatherlands, 16.2 yrs f/u, 47 yrs, 54% W	406/547 (n/N)	24-hour urinary sodium excretion; Q1, Q2, Q3: 2.3, 3.6, 5.8 (g)	Age, sex, race, eGFR, history of DM, kidney disease, CVD, HTN, smoking, 24-hour	Total CVD; 1.73 (1.00, 2.99)

k et al., 2017[29]				urine K, urine Cr, number of antihypertensive agents, the use of RAS blockades	
Prentice et al., 2017[30]	USA, 12 yrs f/u, 70.9 yrs, 100% W	3383/86444 (n/N)	24-hour urinary sodium excretion; 2566.6 (mg)	Age, race, education, family history of premature CVD, smoking, DM, statin, aspirin use, HRT, PA	Total CVD; 1.06 (0.92, 1.23)
Kieneker et al., 2018[31]	Neatherlands, 12.5 yrs f/u, 49.2 yrs, 51.4% W	Q1, Q2, Q3, Q4, Q5 (n/PY): 57/16272, 49/16515, 33/16774, 25/16720, 19/16908	24-hour urinary sodium excretion; Q1, Q2, Q3, Q4, Q5: 83, 110, 132, 159, 209 (mmol)	Age, sex, height, weight, race, smoking, drinking, education, DM, TC/HDL-C, urine K, Mg, Cr, Alb, eGFR	Stroke; Q1, Q2, Q3, Q4, Q5: 1.45 (0.92, 2.29), 1.13 (0.71, 1.79), 1.00 (reference), 1.04 (0.64, 1.71), 0.81 (0.46, 1.41)
Lelli et al., 2018[32]	Italy, 9 yrs f/u, 74.5 yrs, 55.4% W	169/7009 (n/PY)	24-hour urinary sodium excretion; Q1, Q2, Q3: 3.70, 5.55, 7.04 (g)	Age, sex, education, CKD-EPI, SBP, HTN, DM, BMI, energy intake, BW, antihypertensive agents, diuretics use	Total CVD; 0.96 (0.90, 1.02)
Liu et al., 2018[33]	USA, 18.6yrs f/u, Q1,Q2,Q3: 46.2, 46.7, 46.4 yrs; 55.7, 52.2, 47.7 % W	Q1, Q2, Q3 (n/PY): 12/5430, 23/5395, 46/5255	24-hour dietary recall; Q1, Q2, Q3: 35.1, 54.5, 84.1 (mmol/8-hour)	Age, sex, area, work, smoking, drinking, TC, FBG, BMI, region, education, PA, percentage of energy from saturated fat, SBP, antihypertensive agents	Total CVD; Q1, Q2, Q3: 1.00 (reference), 1.66 (0.79, 3.47), 3.04 (1.46, 6.34)
Mente et al., 2018[34]	18 countries [b], 8.1 yrs f/u, 50 yrs, 57.9% W	3543/95767 (n/N)	24-hour urinary sodium excretion; Q1, Q2, Q3: 4.04, 4.70, 5.75 (g)	Age, sex, BMI, education, drinking, smoking, DM, country, PA, anti-	Total CVD; Q1, Q2, Q3: -1.00 (-2.00, 0.01), 0.24 (-2.12, 2.61), 0.37 (-0.03, 0.78)

				hypertensive agents, statin use	(Change in event rates for specific outcomes per 1000 years for each 1 gram increase in sodium intake)
Kondo et al., 2019[35]	Japan, 29 yrs f/u, 50 yrs, 56.1% W	Low, high (n/PY): 57/10350, 1013/213420	3-day dietary records; Low: M: <8 (g), W <7 (g); high: M ≥8, W ≥7(g)	Age, sex, smoking, drinking, energy intake	CVD mortality; 1.35 (1.02, 1.79)
O'Donnell et al., 2019[36]	18 countries[b], 29 yrs f/u, 51 yrs, 57.2% W	Q1, Q2, Q3, Q4, Q5, Q6 (n/N): 949/11002, 1562/21417, 1816/26012, 1640/21093, 964/12458, 953/11218	24-hour urinary sodium excretion; Q1, Q2, Q3, Q4, Q5, Q6 ≤3, 3, 3.99, 4, 4.99, 5, 5.99, 6, 6.99, ≥7 (g):	Age, sex, education, drinking, smoking, DM, BMI, PA, history of CVD, cancer, TB, CVD agents, cancer, HIV	Total CVD; Q1, Q2, Q3, Q4, Q5, Q6: 1.16 (1.03, 1.31), 1.04 (0.94, 1.15), 1.00 (reference), 1.08 (0.98, 1.19), 1.06 (0.95, 1.18), 1.12 (0.99, 1.25)

Abbreviations: AER, albumin excretion rate; Alb, albumin; BMI, body mass index; BP, blood pressure; BW, body weight; C, control; Ca, calcium; CAD, coronary artery disease; CCR, creatinine clearance rate; CHD, Coronary Heart Disease; CKD-EPI, chronic kidney disease epidemiology collaboration; Cr, creatinine; CVD, cardiovascular disease; DBP, diastolic blood pressure; DM, diabetes mellitus; eGFR, estimated glomerular filtration rate; EKG, electrocardiogram; FBG, fasting blood glucose; f/u, follow up; Hb, hemoglobin; HbA1c, glycated hemoglobin; HDL-C, high-density lipoprotein-cholesterol; HIV, Human immunodeficiency virus; HR, heart rate; HRT, hormone replacement therapy; HTN, hypertension; I, intervention; K, potassium; Kt/V, a number used to quantify hemodialysis and peritoneal dialysis treatment adequacy; LDL-C, low-density lipoprotein-cholesterol; LDL-C/HDL-C, low-density lipoprotein-cholesterol to high-density lipoprotein-cholesterol ratio; LVH, left ventricular hypertrophy; M, men; Map, mean arterial pressure; Mg, magnesium; N, numbers; P, phosphate; PA, physical activity; PRA, plasma renin activity; PY, person-years; RAS, renin-angiotensin system; SBP, systolic blood pressure; TB, tuberculosis; TC, cholesterol; TC/HDL-C, total cholesterol to high-density lipoprotein-cholesterol ratio; TG, triglycerides; VHD, valvular heart disease; W, Women; WC, waist circumference

a: Argentina, Bangladesh, Brazil, Canada, Chile, China, Colombia, India, Iran, Malaysia, Pakistan, Poland, South Africa, Sweden, Turkey, United Arab Emirates, and Zimbabwe

b: Argentina, Bangladesh, Brazil, Canada, Chile, China, Colombia, India, Iran, Malaysia, Pakistan, Palestinian territories occupied by Israel, Poland, South

Africa, Sweden, Turkey, United Arab Emirates and Zimbabwe

Table S2. Newcastle–Ottawa scale for assessment of quality of included cohort studies.

Study	Selection				Comparability		Outcome			Final score
	Representativeness of the exposure	Selection of the non-exposed	Ascertainment of exposure	Outcome not present at start	Main factor	Additional factor	Assessment	Follow-up length	Adequacy of follow-up	
Acceptable (★)	a. Representative of general adult population in community<br>b. Somewhat representative (being at risk of CVD)	Drawn from the same community as exposed cohort	a. Secure records<br>b. Structured interview	a. Outcome not present at start<br>b. Excluded when analysis	a. At least for age and sex<br>b. Adjusted age and stratified by sex<br>c. Single sex	Adjusted any other variables	a. Independent blind assessment<br>b. Record linkage	Follow-up > 5 years	a. Follow-up completed<br>b. Small subjects lost (<20%)<br>c. Lost subjects unlikely to introduce bias	
Kagan et al., 1985[1]	★[a]	★	★[b]	★[a]	★[c]	-	★[b]	★	★[c]	8
Hu et al., 1992[2]	★[a]	★	-	★[a]	-	-	★[a]	-	★[c]	5
Alderman et al., 1998[3]	★[a]	★	★[b]	-	★[a]	★	★[b]	★	★[b]	8
He et al., 1999[4]	★[a]	★	★[b]	★[a]	★[a]	★	★[b]	★	-	8
Tuomilehto et al., 2001[5]	★[a]	★	★[a]	★[b]	★[b]	★	★[b]	★	★[c]	9
He et al., 2002[6]	★[a]	★	★[b]	-	★[a]	★	★[b]	★	★[b]	8
Nagata et al., 2004[7]	★[a]	★	★[b]	★[b]	★[b]	★	★[b]	★	★[c]	9
Cohen et al., 2006[8]	★[a]	★	★[b]	★[a]	★[a]	★	★[b]	★	★[c]	9
Cook et al., 2007[9]	★[b].	★	★[a]	★[b]	★[a]	★	★[b]	★	★[b]	9
Geleijnse et al., 2007[10]	★[a]	★	★[a]	★[b]	★[a]	★	★[a]	★	★[b]	9

Cohen et al., 2008[11]	★[a]	★	★[b]	★[a]	★[a]	★	★[b].	★	★[c]	9
Larsson et al., 2008[12]	★[b]	★	★[b]	-	★[c]	★	★[b]	★	★[b]	8
Umesawa et al., 2008[13]	★[a]	★	★[b]	★[a]	★[a]	★	★[b]	★	★[c]	9
Dong et al ., 2010[14]	-	★	★[b]	-	★[a]	★	★[b]	-	★[c]	6
Takachi et al., 2010[15]	★[a]	★	★[b]	★[b]	★[a]	★	★[b]	★	★[b]	9
Yang et al., 2011[16]	★[a]	★	★[b]	★[a]	-	★	★[b]	★	★[c]	8
Gardener et al., 2012[17]	★[a]	★	★[b]	-	★[a]	★	★[b]	★	★[c]	8
Ikehara et al., 2012[18]	★[a]	★	-	★[b]	★[b]	★	★[b]	★	-	7
Cook et al., 2014[19]	★[a]	★	★[a]	-	★	★	★[b]	★	-	7
Horikawa et al., 2014[20]	★[b]	★	★[b]	★[b]	★[a]	★	★[a]	★	-	8
Joosten et al., 2014[21]	★[a]	★	★[a]	-	★[a]	★	★[b]	★	★[c]	8
O'donnell et al., 2014[22]	★[a]	★	★[a]	-	★[a]	★	★[a]	-	★[b]	7
Pfister et al., 2014[23]	★[a]	★	-	★[b]	★[b]	★	-	★	-	6
Kalogeropouloset al., 2015[24]	★[a]	★	★[b]	-	★[a]	★	★[b]	★	★[c]	8
Singer et al., 2015[25]	★[b]	★	★[a]	-	★[a]	★	★[b]	★	★[c]	8
Lamelas et al., 2016[26]	★[a]	★	★[a]	-	★[a]	★	★[a]	-	★[b]	7
Mills et al., 2016[27]	★[b]	★	★[a]	-	★[a]	★	★[b]	★	★[c]	8
Polonia et al., 2016[28]	★[b]	★	★[a]	★[a]	-	★	★[a]	★	-	7
Olde Engberink et al., 2017[29]	★[a]	★	★[a]	-	★[a]	★	★[b]	★	-	7
Prentice et al., 2017[30]	★[b]	★	★[a]	★[a]	★[c]	★	★[a]	★	★[b]	9
Kieneker et al., 2018[31]	★[a]	★	★[a]	★[b]	★[a]	★	★[b]	★	★[c]	9
Lelli et al., 2018[32]	★[b]	★	★[a]	★[a]	★[a]	★	★[a]	★	-	8

Liu et al., 2018[33]	★[a]	★	★[b]	★[b]	★[a]	★	★[b]	★	★[b]	9
Mente et al., 2018[34]	★[a]	★	★[a]	-	★[a]	★	★[a]	★	★[b]	8
Kondo et al., 2019[35]	★[a]	★	★[b]	★[b]	★[a]	★	★[b]	★	★[c]	9
O'Donnell et al., 2019[36]	★[a]	★	★[a]	-	★[a]	★	★[b]	★	★[b]	8

Abbreviation: CVD, cardiovascular disease

Supplemental References. References for Table S1 and Table S2.

1. Kagan, A.; Popper, J.S.; Rhoads, G.G.; Yano, K. Dietary and other risk factors for stroke in Hawaiian Japanese men. *Stroke* **1985**, *16*, 390–396, doi:10.1161/01.str.16.3.390.
2. Hu, H.H.; Sheng, W.Y.; Chu, F.L.; Lan, C.F.; Chiang, B.N. Incidence of stroke in Taiwan. *Stroke* **1992**, *23*, 1237–1241, doi:10.1161/01.str.23.9.1237.
3. Alderman, M.H.; Cohen, H.; Madhavan, S. Dietary sodium intake and mortality: The National Health and Nutrition Examination Survey (NHANES I). *Lancet* **1998**, *351*, 781–785, doi:10.1016/s0140-6736(97)09092-2.
4. He, J.; Ogden, L.G.; Vupputuri, S.; Bazzano, L.A.; Loria, C.; Whelton, P.K. Dietary sodium intake and subsequent risk of cardiovascular disease in overweight adults. *J. Am. Med Assoc.* **1999**, *282*, 2027–2034, doi:10.1001/jama.282.21.2027.
5. Tuomilehto, J.; Jousilahti, P.; Rastenyte, D.; Moltchanov, V.; Tanskanen, A.; Pietinen, P.; Nissinen, A. Urinary sodium excretion and cardiovascular mortality in Finland: A prospective study. *Lancet* **2001**, *357*, 848–851, doi:10.1016/s0140-6736(00)04199-4.
6. He, J.; Ogden, L.G.; Bazzano, L.A.; Vupputuri, S.; Loria, C.; Whelton, P.K. Dietary sodium intake and incidence of congestive heart failure in overweight US men and women: First National Health and Nutrition Examination Survey Epidemiologic Follow-up Study. *Arch. Intern. Med.* **2002**, *162*, 1619–1624, doi:10.1001/archinte.162.14.1619.
7. Nagata, C.; Takatsuka, N.; Shimizu, N.; Shimizu, H. Sodium intake and risk of death from stroke in Japanese men and women. *Stroke* **2004**, *35*, 1543–1547, doi:10.1161/01.STR.0000130425.50441.b0.
8. Cohen, H.W.; Hailpern, S.M.; Fang, J.; Alderman, M.H. Sodium intake and mortality in the NHANES II follow-up study. *Am. J. Med.* **2006**, *119*, 275.e7–275.e14, doi:10.1016/j.amjmed.2005.10.042.
9. Cook, N.R.; Cutler, J.A.; Obarzanek, E.; Buring, J.E.; Rexrode, K.M.; Kumanyika, S.K.; Appel, L.J.; Whelton, P.K. Long term effects of dietary sodium reduction on cardiovascular disease outcomes: Observational follow-up of the trials of hypertension prevention (TOHP). *BMJ* **2007**, *334*, 885–888, doi:10.1136/bmj.39147.604896.55.
10. Geleijnse, J.M.; Witteman, J.C.M.; Stijnen, T.; Kloos, M.W.; Hofman, A.; Grobbee, D.E. Sodium and potassium intake and risk of cardiovascular events and all-cause mortality: The Rotterdam Study. *Eur. J. Epidemiol.* **2007**, *22*, 763–770, doi:10.1007/s10654-007-9186-2.
11. Cohen, H.W.; Hailpern, S.M.; Alderman, M.H. Sodium intake and mortality follow-up in the Third National Health and Nutrition Examination Survey (NHANES III). *J. Gen. Intern. Med.* **2008**, *23*, 1297–1302, doi:10.1007/s11606-008-0645-6.
12. Larsson, S.C.; Virtanen, M.J.; Mars, M.; Männistö, S.; Pietinen, P.; Albanes, D.; Virtamo, J. Magnesium, calcium, potassium, and sodium intakes and risk of stroke in male smokers. *Arch. Intern. Med.* **2008**, *168*, 459–465, doi:10.1001/archinte.168.5.459.
13. Umesawa, M.; Iso, H.; Date, C.; Yamamoto, A.; Toyoshima, H.; Watanabe, Y.; Kikuchi, S.; Koizumi, A.; Kondo, T.; Inaba, Y.; et al. Relations between dietary sodium and potassium intakes and mortality from cardiovascular disease: The Japan Collaborative Cohort study for evaluation of cancer risks. *Am. J. Clin. Nutr.* **2008**, *88*, 195–202.
14. Dong, J.; Li, Y.; Yang, Z.; Luo, J. Low dietary sodium intake increases the death risk in peritoneal dialysis. *Clin. J. Am. Soc. Nephrol.* **2010**, *5*, 240–247, doi:10.2215/cjn.05410709.
15. Takachi, R.; Inoue, M.; Shimazu, T.; Sasazuki, S.; Ishihara, J.; Sawada, N.; Yamaji, T.; Iwasaki, M.; Iso, H.; Tsubono, Y.; et al. Consumption of sodium and salted foods in relation to cancer and cardiovascular disease: The Japan public health center-based prospective study. *Am. J. Clin. Nutr.* **2010**, *91*, 456–464, doi:10.3945/ajcn.2009.28587.
16. Yang, Q.; Liu, T.; Kuklina, E.V.; Flanders, W.D.; Hong, Y.; Gillespie, C.; Chang, M.H.; Gwinn, M.; Dowling, N.; Khoury, M.J.; et al. Sodium and potassium intake and mortality among US adults: Prospective data from the Third National Health and Nutrition Examination Survey. *Arch. Intern. Med.* **2011**, *171*, 1183–1191, doi:10.1001/archinternmed.2011.257.
17. Gardener, H.; Rundek, T.; Wright, C.B.; Elkind, M.S.V.; Sacco, R.L. Dietary sodium and risk of stroke in the Northern Manhattan Study. *Stroke* **2012**, *43*, 1200–1205, doi:10.1161/STROKEAHA.111.641043.

18. Ikehara, S.; Iso, H.; Date, C.; Kikuchi, S.; Watanabe, Y.; Inaba, Y.; Tamakoshi, A. Salt preference and mortality from stroke and coronary heart disease for Japanese men and women: The JACC study. *Prev. Med.* **2012**, *54*, 32–37, doi:10.1016/j.ypmed.2011.10.013.
19. Cook, N.R.; Appel, L.J.; Whelton, P.K. Lower levels of sodium intake and reduced cardiovascular risk. *Circulation* **2014**, *129*, 981–989, doi:10.1161/circulationaha.113.006032.
20. Horikawa, C.; Yoshimura, Y.; Kamada, C.; Tanaka, S.; Tanaka, S.; Hanyu, O.; Araki, A.; Ito, H.; Tanaka, A.; Ohashi, Y.; et al. Dietary sodium intake and incidence of diabetes complications in Japanese patients with type 2 diabetes: Analysis of the Japan diabetes complications study (JDCS). *J. Clin. Endocrinol. Metab.* **2014**, *99*, 3635–3643, doi:10.1210/jc.2013-4315.
21. Joosten, M.M.; Gansevoort, R.T.; Mukamal, K.J.; Heerspink, H.J.L.; Geleijnse, J.M.; Feskens, E.J.M.; Navis, G.; Bakker, S.J.L. Sodium excretion and risk of developing coronary heart disease. *Circulation* **2014**, *129*, 1121–1128, doi:10.1161/CIRCULATIONAHA.113.004290.
22. O'Donnell, M.; Mente, A.; Rangarajan, S.; McQueen, M.J.; Wang, X.; Liu, L.; Yan, H.; Lee, S.F.; Mony, P.; Devanath, A.; et al. Urinary sodium and potassium excretion, mortality, and cardiovascular events. *N. Engl. J. Med.* **2014**, *371*, 612–623, doi:10.1056/NEJMoa1311889.
23. Pfister, R.; Michels, G.; Sharp, S.J.; Luben, R.; Wareham, N.J.; Khaw, K.T. Estimated urinary sodium excretion and risk of heart failure in men and women in the EPIC-Norfolk study. *Eur. J. Heart Fail.* **2014**, *16*, 394–402, doi:10.1002/ejhf.56.
24. Kalogeropoulos, A.P.; Georgiopoulou, V.V.; Murphy, R.A.; Newman, A.B.; Bauer, D.C.; Harris, T.B.; Yang, Z.; Applegate, W.B.; Kritchevsky, S.B. Dietary sodium content, mortality, and risk for cardiovascular events in older adults: The health, aging, and body composition (Health ABC) study. *JAMA Intern. Med.* **2015**, *175*, 410–419, doi:10.1001/jamainternmed.2014.6278.
25. Singer, P.; Cohen, H.; Alderman, M. Assessing the associations of sodium intake with long-term all-cause and cardiovascular mortality in a hypertensive cohort. *Am. J. Hypertens.* **2015**, *28*, 335–342, doi:10.1093/ajh/hpu141.
26. Lamelas, P.M.; Mente, A.; Diaz, R.; Orlandini, A.; Avezum, A.; Oliveira, G.; Lanas, F.; Seron, P.; Lopez-Jaramillo, P.; Camacho-Lopez, P.; et al. Association of urinary sodium excretion with blood pressure and cardiovascular clinical events in 17,033 Latin Americans. *Am. J. Hypertens.* **2016**, *29*, 796–805, doi:10.1093/ajh/hpv195.
27. Mills, K.T.; Chen, J.; Yang, W.; Appel, L.J.; Kusek, J.W.; Alper, A.; Delafontaine, P.; Keane, M.G.; Mohler, E.; Ojo, A.; et al. Sodium excretion and the risk of cardiovascular disease in patients with chronic kidney disease. *JAMA J. Am. Med Assoc.* **2016**, *315*, 2200–2210, doi:10.1001/jama.2016.4447.
28. Polonia, J.; Monteiro, J.; Almeida, J.; Silva, J.A.; Bertoquini, S. High salt intake is associated with a higher risk of cardiovascular events: A 7.2-year evaluation of a cohort of hypertensive patients. *Blood Press. Monit.* **2016**, *21*, 301–306, doi:10.1097/mbp.0000000000000205.
29. Olde Engberink, R.H.G.; Van Den Hoek, T.C.; Van Noordenne, N.D.; Van Den Born, B.J.H.; Peters-Sengers, H.; Vogt, L. Use of a single baseline versus multiyear 24-hour urine collection for estimation of long-term sodium intake and associated cardiovascular and renal risk. *Circulation* **2017**, *136*, 917–926, doi:10.1161/CIRCULATIONAHA.117.029028.
30. Prentice, R.L.; Huang, Y.; Neuhouser, M.L.; Manson, J.E.; Mossavar-Rahmani, Y.; Thomas, F.; Tinker, L.F.; Allison, M.; Johnson, K.C.; Wassertheil-Smoller, S.; et al. Associations of Biomarker-Calibrated Sodium and Potassium Intakes with Cardiovascular Disease Risk among Postmenopausal Women. *Am. J. Epidemiol.* **2017**, *186*, 1035–1043, doi:10.1093/aje/kwx238.
31. Kieneker, L.M.; Eisenga, M.F.; Gansevoort, R.T.; de Boer, R.A.; Navis, G.; Dullaart, R.P.F.; Joosten, M.M.; Bakker, S.J.L. Association of Low Urinary Sodium Excretion with Increased Risk of Stroke. *Mayo Clin. Proc.* **2018**, *93*, 1803–1809, doi:10.1016/j.mayocp.2018.05.028.
32. Lelli, D.; Antonelli-Incalzi, R.; Bandinelli, S.; Ferrucci, L.; Pedone, C. Association between sodium excretion and cardiovascular disease and mortality in the elderly: A cohort study. *J. Am. Med Dir. Assoc.* **2018**, *19*, 229–234, doi:10.1016/j.jamda.2017.09.004.

33. Liu, H.; Gao, X.; Zhou, L.; Wu, Y.; Li, Y.; Mai, J.; Nie, Z.; Wu, Y.; Liu, X.; Zhao, L. Urinary sodium excretion and risk of cardiovascular disease in the Chinese population: A prospective study. *Hypertens. Res.* **2018**, *41*, 849–855, doi:10.1038/s41440-018-0091-8.
34. Mente, A.; O'Donnell, M.; Rangarajan, S.; McQueen, M.; Dagenais, G.; Wielgosz, A.; Lear, S.; Ah, S.T.L.; Wei, L.; Diaz, R.; et al. Urinary sodium excretion, blood pressure, cardiovascular disease, and mortality: A community-level prospective epidemiological cohort study. *Lancet* **2018**, *392*, 496–506, doi:10.1016/S0140-6736(18)31376-X.
35. Kondo, K.; Miura, K.; Tanaka-Mizuno, S.; Kadota, A.; Arima, H.; Okuda, N.; Fujiyoshi, A.; Miyagawa, N.; Yoshita, K.; Okamura, T.; et al. Cardiovascular risk assessment chart by dietary factors in Japan—NIPPON DATA80. *Circ. J.* **2019**, *83*, 1254–1260, doi:10.1253/circj.CJ-18-1002.
36. O'Donnell, M.; Mente, A.; Rangarajan, S.; McQueen, M.J.; O'Leary, N.; Yin, L.; Liu, X.; Swaminathan, S.; Khatib, R.; Rosengren, A.; et al. Joint association of urinary sodium and potassium excretion with cardiovascular events and mortality: Prospective cohort study. *BMJ* **2019**, *364*, l772, doi:10.1136/bmj.l772.
